# Bipolar Cell Type-Specific Expression and Conductance of Alpha-7 Nicotinic Acetylcholine Receptors in the Mouse Retina

**DOI:** 10.1167/iovs.18-25753

**Published:** 2019-04

**Authors:** Leo M. Hall, Chase B. Hellmer, Christina C. Koehler, Tomomi Ichinose

**Affiliations:** Department of Ophthalmology, Visual and Anatomical Sciences, Wayne State University School of Medicine, Detroit, Michigan, United States

**Keywords:** acetylcholine receptor, immunohistochemistry, patch clamp, mouse retina

## Abstract

**Purpose:**

Motion detection is performed by a unique neural network in the mouse retina. Starburst amacrine cells (SACs), which release acetylcholine and gamma-aminobutyric acid (GABA) into the network, are key neurons in the motion detection pathway. Although GABA contributions to the network have been extensively studied, the role of acetylcholine is minimally understood. Acetylcholine receptors are present in a subset of bipolar, amacrine, and ganglion cells. We focused on α7-nicotinic acetylcholine receptor (α7-nAChR) expression in bipolar cells, and investigated which types of bipolar cells possess α7-nAChRs.

**Methods:**

Retinal slice sections were prepared from C57BL/6J and Gus8.4-GFP mice. Specific expression of α7-nAChRs in bipolar cells was examined using α-bungarotoxin (αBgTx)-conjugated Alexa dyes co-labeled with specific bipolar cell markers. Whole-cell recordings were conducted from bipolar cells in retinal slice sections. A selective α7-nAChR agonist, PNU282987, was applied by a puff and responses were recorded.

**Results:**

αBgTx fluorescence was observed primarily in bipolar cell somas. We found that α7-nAChRs were expressed by the majority of type 1, 2, 4, and 7 bipolar cells. Whole-cell recordings revealed that type 2 and 7 bipolar cells depolarized by PNU application. In contrast, α7-nAChRs were not detected in most of type 3, 5, 6, and rod bipolar cells.

**Conclusions:**

We found that α7-nAChRs are present in bipolar cells in a type-specific manner. Because these bipolar cells provide synaptic inputs to SACs and direction selective ganglion cells, α7-nAChRs may play a role in direction selectivity by modulating these bipolar cells' outputs.

Visual processing begins in the retina, where images are captured by photoreceptors and are processed by the following retinal second- and third-order neurons: bipolar, amacrine, and ganglion cells. Each of these neurons consists of multiple subsets, including approximately 15 types of bipolar cells,^1^–^5^ more than 20 types of amacrine cells,^6^,^7^ and 15 to more than 30 types of ganglion cells.^8^–^11^ These diverse types of neurons are thought to form parallel neural networks that encode distinct image features.

Parallel neural networks are initiated by a variety of second-order bipolar cell types. Different types of bipolar cells have unique signaling characteristics^12^–^15^ and connect to different postsynaptic partners.^3^ Additionally, the output of each bipolar cell can be further tuned by inhibition, allowing for a greater diversity of information output.^16^,^17^ Such a wealth of factors underlies the phenomena of parallel processing, whereby unique circuits initiated by bipolar cells carry distinct information, such as color, contrast, or possibly even motion.

Visual motion detection is a vital function in animal survival. Certain retinal neurons are known contributors to retinal motion detection. One such example is the multiple types of direction selective ganglion cells (DSGCs), which are sensitive to objects moving in a particular direction.^18^,^19^ DSGC motion tracking depends on synaptic inputs from starburst amacrine cells (SACs),^20^,^21^ another neuronal class participating in retinal motion detection. SACs receive synaptic inputs from bipolar cells and release both gamma-aminobutyric acid (GABA) and acetylcholine to postsynaptic neurons, which also exhibit direction selectivity.^22^–^24^ SACs release GABA on to DSGCs in response to light moving in the SAC's preferred direction, which induces unidirectional excitation in these ganglion cells.^21^,^25^–^27^ Although GABA release from SACs to DSGCs has been rigorously studied, the role of acetylcholine remains unclear.

Receptors for acetylcholine are broadly distributed in the central nervous system, including the retina. They are classified into two receptor classes as follows: nicotinic and muscarinic receptors. Nicotinic receptors are cationic channels that induce excitation in cells. In contrast, muscarinic receptors are G protein–coupled receptors that signal via G_q_ or G_i_ subunits. Nicotinic acetylcholine receptors are expressed by DSGCs, which receive cholinergic input directly from SACs.^28^–^30^ Nicotinic and muscarinic receptors are also present in subsets of bipolar, amacrine, and other ganglion cells in the mammalian retina.^28^,^31^–^33^ Given that SACs are the only known source of acetylcholine in the retina, these acetylcholine receptor–expressing neurons may also contribute to motion computations.

We focused on α7 nicotinic acetylcholine receptors (α7-nAChRs) because these receptors distribute broadly in the central nervous system, induce depolarization with high calcium permeability, modulate presynaptic transmitter release, and exhibit a high sensitivity for certain toxins, such as a-bungarotoxin (aBgTx).^34^–^38^ We examined α7-nAChR-specific expression among multiple bipolar cell types using immunostaining methods. Additionally, α7-nAChR conductance was examined using patch-clamp methods. In the present work, we demonstrate that α7-nAChRs are expressed by bipolar cells in a type-specific manner.

## Methods

### Retinal Preparation

Animal protocols were approved by the Institutional Animal Care and Use Committee at Wayne State University. Experiments were performed in accordance with the ARVO Statement for the Use of Animals in Ophthalmic and Visual Research. We used wild-type mice (C57BL/6J; Jackson Laboratory, Bar Harbor, ME, USA) and Gus8.4-GFP mice (gift from Robert Margolskee^39^). The experimental techniques were similar to those described previously.^15^,^40^ Briefly, mice (28–60 days of age) were euthanized using carbon dioxide and cervical dislocation. Using a dissecting microscope, the retina was isolated, the retinal slab placed onto a piece of filter membrane (HABG01300; Millipore, Burlington, MA, USA) and cut into slice preparations (250-μm thick) using a hand-made chopper. Retinal dissections were performed in HEPES-buffered solution containing the following (in mM): 115 NaCl, 2.5 KCl, 2.5 CaCl_2_, 1.0 MgCl_2_, 10 HEPES, 28 glucose, adjusted to pH 7.38 by NaOH. The dissection medium was cooled and continuously oxygenated. The retinal preparations were stored in an oxygenated dark box at room temperature.

### Whole-Cell Recordings and Puff Application

Whole-cell patch recordings were made from bipolar cell somas in retinal slices by viewing them with an upright microscope (Slicescope Pro 2000; Scientifica, Uckfield, East Sussex, UK) equipped with a charged-couple device camera (Retiga-2000R; Q-Imaging, Surrey, BC, Canada). Recordings were performed in Ames' medium buffered with NaHCO_3_ (Sigma, St. Louis, MO, USA), which was continuously bubbled with 95% O_2_ and 5% CO_2_; the pH was 7.4 at 30°C. Electrodes were pulled from borosilicate glass (1B150F-4; World Precision Instruments, Sarasota, FL, USA) with a P1000 Micropipette Puller (Sutter Instruments, Novato, CA, USA) and had resistances of 8 to 12 MΩ. The intracellular solution contained the following (in mM): 111 K-gluconate, 1.0 CaCl_2_, 10 HEPES, 10 EGTA, 10 NaCl, 1.0 MgCl_2_, 5 ATP-Mg, and 1.0 GTP-Na, adjusted to pH 7.2 with CsOH. Liquid junction potentials were corrected after each recording. Clampex and Multi Clamp 700B (Molecular Devices, San Jose, CA, USA) were used to acquire data and to control puff application using a Picospritzer III (Parker Hannifin, Cleveland, OH, USA). A selective α7-nAChR agonist, PNU282987 (30 μM; Tocris, Bristol, UK) was puffed onto recording bipolar cell axon terminals. The data were digitized and stored with a computer using Axon Digidata 1440A (Molecular Devices). Responses were filtered at 1 kHz with the four-pole Bessel filter and sampled at 2 to 5 kHz.

A fluorescent dye, sulforhodamine B (0.005%; Sigma), and Neurobiotin (NB; 0.5%; Vector Lab, Burlingame, CA, USA) were included in the recording pipette; these dyes did not affect the physiological recordings.^15^ Sulforhodamine B images were observed after physiological recordings. To visualize NB staining, the slice preparation was fixed with 4% paraformaldehyde for 30 minutes, incubated with streptavidin-conjugated Alexa 488 (1:200; Life Technologies, Carlsbad, CA, USA) and anti-ChAT antibody (1:200; Millipore) overnight, and then incubated with the secondary antibody for 2 hours at room temperature. The preparation was viewed with a confocal microscope (TCS SP8; Leica, Wetzlar, Hesse, Germany) using a water-immersion, ×63 objective. Bipolar cell types were determined based on previous references.^1^,^15^

### Immunohistochemistry

Using live retinal slice sections, either αBgTx -conjugated Alexa 488 or 555 (1:100) was applied for 1 hour, followed by several rinses with HEPES buffer solution, and fixation using 4% paraformaldehyde for 30 minutes. After several washes in 0.1 M PBS, sections were blocked in a solution containing 10% normal donkey serum (NDS) and 0.5% Triton X-100 in PBS for 1 hour at room temperature. Primary antibodies (Table 1) were diluted in 3% NDS and 0.5% Triton X-100 in PBS. Sections were incubated with the primary antibody overnight at room temperature, and then incubated with a secondary antibody conjugated with Alexa dyes (Thermo Fisher Scientific, Waltham, MA, USA) for 2 hours. The preparation was viewed with a confocal microscope (TCS SP8; Leica) using ×63 water immersion objectives. The z-step for stack images was 0.3 μm.

**Table 1:** Immunohistochemistry Antibodies and Toxins

**Antibody**	**Immunogen**	**Source, Cat. #, Species**	**RRID**	**Dilution**
α-Bungarotoxin 488	α-Subunit of nAchR	Thermo Fisher Scientific, B13422		1:100
α-Bungarotoxin 555	α-Subunit of nAchR	Thermo Fisher Scientific, B35451	AB_2617152	1:100
α7-nAChR (extracellular)	Peptide (C) KELVKNYNPLER, amino acid residues 31–42 of rat nAChR	Alomone (Jerusalem, Israel), ANC-007, Rabbit polyclonal	AB_10659339	1:500
Calsenilin/DREAM, clone 40A5	Full-length GST fusion protein of human Calsenilin	EMD Millipore, 05-756, Mouse monoclonal	AB_2313634	1:1000
ChAT	Human placental enzyme	EMD Millipore, AB144P, Goat polyclonal	AB_2079751	1:200
HCN4 clone N114/10	Fusion protein amino acids 1019-1108 at C-terminus of rat HCN4	NeuroMab (Davis, CA, USA), 75-150, Mouse monoclonal	AB_2248534	1:200
NK3R	Amino acids 410-417 of rat neurokinin3 receptor (NK3R)	Gift from Dr. Hirano, Rabbit polyclonal	AB_2314947	1:700
PKA RIIβ	Amino acids 1-418 of human PKA RIIβ	BD Biosciences (San Jose, CA, USA), 610625, Mouse monoclonal	AB_397957	1:3000
PKC α	Amino acids 645-672 at C-terminus of human PKC α	Santa Cruz Biotechnology (Dallas, TX, USA), sc-8393, Mouse monoclonal	AB_628142	1:500
Synaptotagmin-2	Zebrafish Syt2	ZIRC (Eugene, OR, USA), znp-1, Mouse monoclonal	AB_10013783	1:200

RRID, research resource identifiers; ZIRC, Zebrafish International Resource Center.

### Western Blot

Retinal tissue was lysed by sonication in radioimmunoprecipitation assay buffer with a protease inhibitor cocktail (P8340; Sigma) and was sonicated followed by centrifugation. Total protein samples (40 ug) were run on a 12% SDS-PAGE in Tris-glycine-SDS buffer and then electroblotted onto a nitrocellulose membrane (BioRad, Hercules, CA, USA). After blocking with 5% BSA in Tris-buffered saline containing 0.05% Tween 20 and 5% milk (TBS-T) for 1 hour, the membrane was probed with the α7-nAChR primary antibody (1:1000) or primary antibody (1:1000) incubated with blocking peptide (1:200) for 30 minutes in 3% BSA in TBS-T overnight at 4°C. Then, the membrane was incubated with a horseradish peroxidase secondary antibody (1:2000) diluted in TBS-T with 5% milk at room temperature for 2 hours. Bands were visualized using a Kodak 4000R Pro Molecular Imaging System (Carestream Health Inc., Rochester, NY, USA).

### Data Analysis

Using AutoQuant X3 and Image-Pro Premier 3D software (Media Cybernetics, Rockville, MD, USA), we conducted image deconvolution and three-dimensional alignment. ChAT bands in stack images were realigned and NB-filled bipolar cells were analyzed. We analyzed colocalization of bipolar cell markers with αBgTx fluorescence or α7-nAChR antibody by examining each soma in each digital section (0.3 μm). A stained cell was categorized as a bipolar cell if the cell possesses dendrites.

## Results

We conducted immunohistochemistry to examine α7-nAChR expression in each type of bipolar cell, and patch clamp recordings to examine α7-nAChR conductance in bipolar cells.

### α-BgTx Fluorescence and α7 Antibody Staining

**Figure 1:**
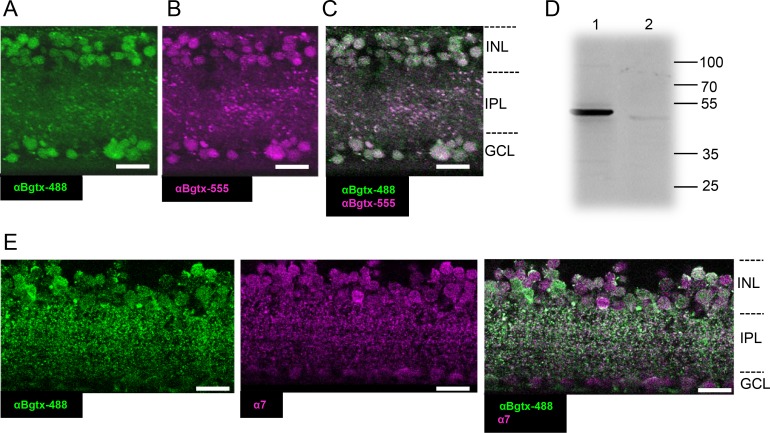
αBgTx-sensitive, α7-nAChRs were expressed in retinal bipolar cells. (A–C) αBgTx-conjugated with Alexa Fluor 488- (green) and Alexa Fluor 555- (magenta) stained retinal neurons, including bipolar cells. When stained together, the two colors colocalized extensively (78/78 cells colocalized, n = 3 mice). (D) The α7-nAChR antibody was verified by Western blots in retinal tissue (lane 1), in which a solid band was seen at approximately 55 kDa and 58 kDa. Retinal tissue was then preincubated with peptide antigen (lane 2) in order to test for antibody specificity. Preincubation treatment led to the removal of the expected approximately 55 kDa band. (E) αBgTx-conjugated Alexa 488-labeled cells (green), which colocalized with α7-nAChR antibody staining (magenta). Scale bar: 15 μm. Images are maximum intensity projections of multiple slice sections. INL, inner nuclear layer; IPL, inner plexiform layer; GCL, ganglion cell layer.

Using retinal slice sections, we labeled α7-nAChR-expressing cells by αBgTx-conjugated Alexa 488 or Alexa 555 application. Many somas in the inner nuclear layer (INL) and ganglion cell layer were labeled (Figs. 1A, 1B), suggesting that a subset of bipolar, amacrine, and ganglion cells express α7-nAChRs. Both Alexa dyes labeled the same sets of cells (Fig. 1C; 78/78 cells colocalized, *n* = 3 mice). αBgTx is highly specific for α7-nAChRs in the mammalian retina^41^,^42^; however, it may also bind to the β3 GABA_A_ receptor subunit.^43^,^44^ Therefore, we used an antibody against α7-nAChRs to investigate whether αBgTx-stained cells are specific to α7-nAChRs. We first authenticated the α7-nAChR antibody (Table 1) by Western blot analysis on retinal tissue. The α7-nAChR antibody demonstrated a solid band at approximately 55 kDa, consistent with previous reports (lane 1, Fig. 1D).^32^,^45^–^47^ We also saw an additional band at approximately 58 kDa, which matches the known molecular weight of the splice variant α7-2.^46^ When preabsorbing the antibody with the peptide antigen, no bands were observed, which authenticated the antibody (lane 2, Fig. 1D). Then, we co-applied αBgTx and the α7-nAChR antibody to retinal slice preparations (*n* = 3 mice, Fig. 1E). Almost all cells demonstrating αBgTx fluorescence also expressed α7-nAChRs (192/194, 99.0%), confirming that αBgTx cells express α7-nAChRs. We used αBgTx conjugated with Alexa dyes for subsequent studies.

### OFF Transient Bipolar Cells

**Figure 2:**
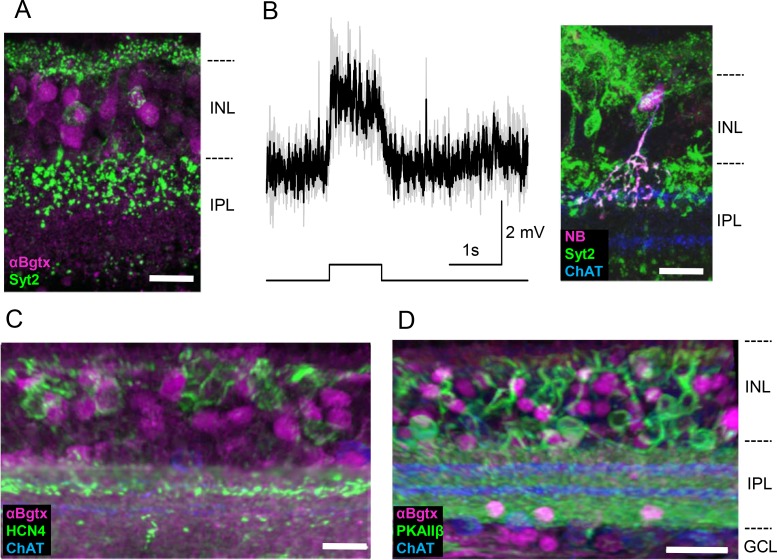
Type 2, but not type 3, OFF bipolar cells possessed α7-nAChRs. (A) Type 2 bipolar cells were identified with Syt2 (green), which showed αBgTx fluorescence (magenta, 32/39 cells). (B) Type 2 bipolar cells depolarized in response to PNU282987 puff (left). Individual traces shown in gray, and average trace displayed in black. A NB-filled cell depolarized in response to PNU, which was confirmed by Syt2 staining (right). (C) HCN4-labeled type 3a (green), which were partially visualized with αBgTx fluorescence (magenta) (20/89 cells). (D) PKAIIβ-labeled type 3b bipolar cells (green), which were not labeled with αBgTx fluorescence (magenta) (0/15 cells) PKAIIβ-labeled various amacrine cells that localize along the outer border of the INL.^48^ Scale bar: (A–C) 10 μm, (D) 20 μm. Images are maximum intensity projections of multiple slice sections.

Previously, we investigated the physiological signaling properties of each type of bipolar cell and found that some bipolar cells exhibit transient signaling while others exhibit slower, sustained signaling.^14^ We thought that transient bipolar cells might be important for detecting motion stimuli and may express α7-nAChRs. We first examined type 2, 3a, and 3b bipolar cells, which constitute the kinetically transient neurons among the five types of OFF bipolar cells.^14^ OFF bipolar cells are characterized by their axonal ramification processes close to the OFF-ChAT band. We used specific markers for these bipolar cells, including synaptotagmin 2 (Syt2) for type 2, HCN4 for type 3a, and PKAIIβ for type 3b.^2^,^48^ Of Syt2-expressing type 2 bipolar cells, 82% were labeled with αBgTx fluorescence (Fig. 2A, *n* = 5 mice, 32/39 cells were positive). We also conducted whole-cell recordings to examine whether these receptors depolarized the cell after application of a specific α7-nAChR agonist, PNU282987. Type 2 cells depolarized in response to a PNU282987 puff application onto their axon terminals (Fig. 2B, 3.6 ± 0.5 mV, 5/6 cells depolarized). To verify these responses were attributable to direct activation of α7-nAChRs, we applied CoCl_2_ (4 mM) in the bath solution, which eliminates synaptic transmission. After disrupting retinal network effects with CoCl_2,_ we observed that type 2 bipolar cells were still depolarized by PNU application. Taken together, these results indicate that type 2 bipolar cells possess active α7-nAChRs.

In contrast, only 22% of HCN4-labeled type 3a cells colocalized with αBgTx (Fig. 2C, *n* = 4 mice, 20/89 cells were positive) and no PKAIIβ-labeled type 3b cells colocalized with αBgTx fluorescence (Fig. 2D, *n* = 2 mice, 0/15 cells were positive). Whole-cell recordings revealed that both type 3a and 3b cells were unresponsive to PNU282987 puff (*n* = 10 cells, 4 were confirmed type 3a). We therefore concluded that both type 3a and 3b cells do not express functional α7-nAChRs.

### ON Transient Bipolar Cells

**Figure 3:**
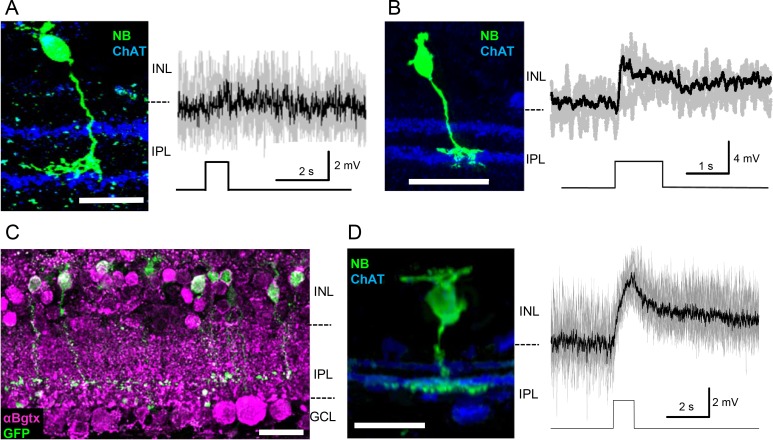
Type 7, but not type 5 possessed α7-nAChRs. (A) The majority of type 5 bipolar cells did not respond to PNU puff (19/22 cells). Individual traces shown in gray, and average trace displayed in black. (B) Three of 22 type 5 cells depolarized in response to PNU puff. They were all with narrow-stratifying axon terminals, possibly a subset of sustained type 5 cells. (C) Gus8.4-GFP-expressing type 7 bipolar cells displayed extensive colocalization with αBgTx fluorescence (55/62 cells). (D) Type 7 cells all depolarized in response to PNU puff (6/6 cells). Recording cells were identified as type 7 by immunoreactivity with ChAT antibody and NB (right). Images are maximum intensity projections of multiple slice sections. Scale bar: (A–D) 10 μm.

Similar to OFF bipolar cells, ON bipolar cells also exhibit either transient or sustained signaling kinetics in a type-dependent manner.^15^ A majority of type 5 and 7 bipolar cells are transient and ramify close to the ON ChAT band. Given this relationship between type 5 and 7 bipolar cell axon terminals and ON-SAC processes, we hypothesized that type 5 and/or 7 cells would respond to a cholinergic stimulus. We used the Gus-GFP mice in order to identify type 7 bipolar cells.^2^,^49^ Type 5 bipolar cells comprise multiple subsets,^2^,^3^,^13^,^50^,^51^ and there are no appropriate markers available for type 5 subsets. Therefore, we conducted whole-cell recordings to examine the physiological properties of α7-nAChRs in these cells. A majority of type 5 bipolar cells did not respond to PNU282987 puff application, indicating that these type 5 cells do not possess α7-nAChRs (Fig. 3A). However, three of 22 cells depolarized in response to PNU (Fig. 3B). Axon terminals of these cells were relatively narrow (12.5 ± 0.3 μm) and were not XBC-like.^3^,^13^ Furthermore, two of three cells were tested with CoCl_2_ application and remained depolarizing in response to PNU, suggesting that a subset of sustained type 5 bipolar cells responded to PNU puff.^15^

In contrast, 89% of GFP-expressing type 7 bipolar cells exhibited αBgTx fluorescence (Fig. 3C, *n* = 5 mice, 55/62 cells were positive). All type 7 bipolar cells depolarized in response to the PNU puff (Fig. 3D, 4.0 ± 0.7 mV, 6/6 cells depolarized). These results indicate that type 7 bipolar cells possess functional α7-nAChRs.

### Sustained Bipolar Cells

**Figure 4:**
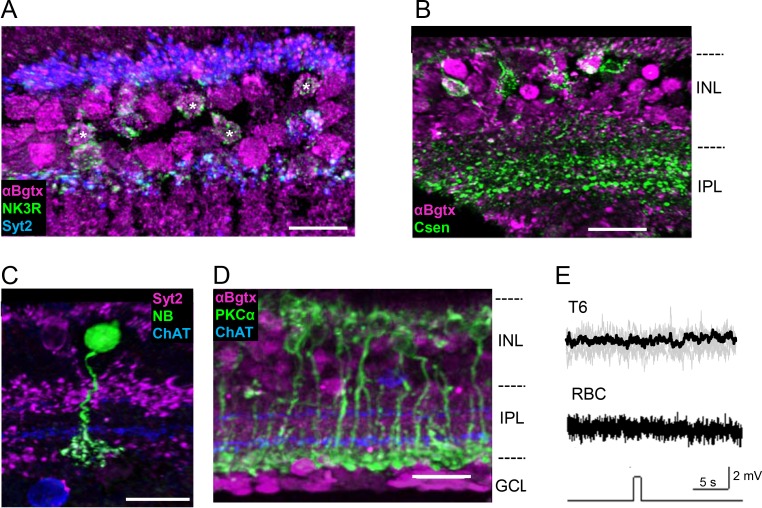
α7-nAChRs in sustained bipolar cells. (A) Using N3KR and Syt2 antibodies, type 1 bipolar cells were identified (asterisk), which were positive for NK3R (green) but negative for Syt2 (blue). αBgTx fluorescence (magenta) labeled a majority of type 1 cells (23/25 cells). (B) Type 4 bipolar cells were identified by Csen (green), which were mostly labeled with αBgTx-fluorescence (magenta, 31/38 cells). (C) Type 6 bipolar cells were recorded by whole-cell configuration. These cells were filled with NB (green) and identified with Syt2 (magenta) axon labeling. (D) RBCs, which were identified with PKC α labeling (green), did not express αBgTx-sensitive α7-nAChRs (magenta). (E) A majority of type 6 cells did not respond to PNU puff (T6). All RBCs recorded did not respond to PNU puff (RBC). Scale bar: (A–C) 10 μm, (D) 20 μm. Images are maximum intensity projections of multiple slice sections.

We examined four different types of sustained bipolar cells, type 1, 4, 6, and rod bipolar cells (RBCs). We used NK3R and calsenilin (Csen) antibodies to label type 1 and 4 OFF bipolar cells, respectively.^52^,^53^ NK3R antibody labels both type 1 and 2 bipolar cells.^53^ We used NK3R and Syt2, a specific type 2 marker, antibodies to identify type 1 cells. A majority of type 1 bipolar cells demonstrated αBgTx fluorescence (Fig. 4A, *n* = 3 mice, 23/25 cells were positive). Similarly, a majority of Csen-expressing, type 4 bipolar cells showed αBgTx fluorescence (Fig. 4B, *n* = 4 mice, 31/38 cells were positive). These results indicated that type 1 and 4 bipolar cells express α7-nAChRs. Type 1 and 4 cells were rarely encountered during patch-clamp recording; therefore, we cannot demonstrate whether these cells are responsive to PNU puff.

Syt2 is a marker for type 6 bipolar cells; however, it labels only type 6 axon terminals, not somas.^2^,^54^ Syt2 is therefore not suitable for detecting α7-nAChR-expression in type 6 cells. Given this information, we performed patch-clamp recordings with NB staining to examine whether type 6 cells possess α7-nAChRs. A majority of type 6 cells did not respond to PNU puff (Fig. 4C, 6/7 cells were negative), suggesting that type 6 cells do not possess α7-nAChRs.

PKCα labels rod bipolar cells (RBCs),^55^ which did not demonstrate αBgTx fluorescence (Fig. 4D, *n* = 2 mice, 65/65 cells were negative). In addition, whole-cell recordings revealed that none of RBCs depolarized in response to PNU application (0/22 cells depolarized). In almost half of cases, RBCs hyperpolarized in response to PNU puff (10/22 hyperpolarized). This response was blocked with CoCl_2_, suggesting that the hyperpolarization was the consequence of surrounding amacrine cell excitation by PNU puff rather than direct responses of RBCs.^56^ These results indicate that RBCs do not possess α7-nAChRs.

We could not investigate α7-nAChRs in type 8 and 9 bipolar cells because the fluorescent marker in clomeleon-1 mice, which highlight type 8 and 9 cells,^2^ fades out over multiple generations.^54^ Furthermore, we did not encounter these cells by patch-clamp recordings because of their low density compared with other bipolar cell subtypes.^2^

Altogether, we found that a subset of bipolar cells express α7-nAChRs, which are summarized in Table 2.

**Table 2:**
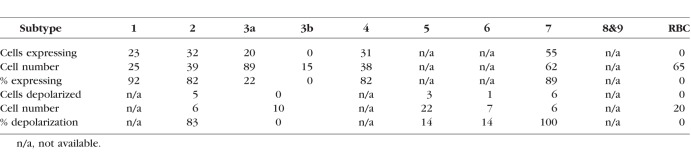
Types of Bipolar Cells Exhibited a7-nAChRs

## Discussion

We examined the α7-nAChR expression in nine types of bipolar cells by immunostaining, and measured physiological activity by measuring conductance of α7-nAChRs. As shown in Table 2, type 2 and 7 bipolar cells exhibited α7-nAChRs expression and conductance, as demonstrated through immunostaining and whole-cell recording, respectively. Additionally, immunostaining revealed that the majority of type 1 and 4 cells showed fluorescence when stained with αBgTx-conjugated Alexa dye, demonstrating that these cells express α7-nAChRs. We therefore conclude that four types of bipolar cells possess α7-nAChRs. In contrast, type 3a, 3b, 5, 6, and RBCs did not exhibit α7-nAChRs. Although a small portion of type 3a cells colocalized with αBgTx fluorescence (Fig. 2C), none of the type 3 cells responded to PNU puff application. The majority of type 5 cells did not respond to PNU puff. However, a small type 5 population responded to PNU puff (3/22 cells), which could represent a subset of type 5 cells.^2^,^3^,^13^,^50^ We ultimately concluded that the majority of type 3a, 3b, 5, 6, and rod bipolar cells do not possess α7-nAChRs.

αBgTx has a high affinity for α7-nAChRs; however, αBgTx also has an affinity for other homomeric α8 or α9 nAChRs.^57^ Although the α8 nAChR isoform is uniquely expressed in avian retina^42^,^58^–^60^ and α9 is mainly expressed in cochlea and vestibular organs,^42^ we cannot rule out the possibility of α8 or α9 nAChR expression in the mouse retina. We therefore conducted PNU application to verify the α7 expression in a subset of bipolar cells.

We demonstrated that both αBgTx-conjugated Alexa dye and the α7 antibody visualize somas as well as axons of bipolar cells, consistent with previous findings from Keyser's group.^32^,^33^ We used live-tissue preparation for αBgTx staining, indicating that α7 nAChRs are expressed from the axons to the somas of bipolar cells. This diffuse expression suggests that either synaptic or paracrine transmission could occur from SACs to bipolar cells, consistent with previous reports.^29^,^30^,^32^ This may explain the expression of α7-nAChRs in type 1 bipolar cells, which do not have an intimate relationship with the cholinergic plexus like other α7-expressing cells.^14^ Expression of α7-nAChR as well as other nicotinic and muscarinic receptors have also been shown in subsets of bipolar cells.^4^,^32^,^33^,^61^ In the present study, we examined the bipolar cell types that possess α7-nAChRs to elucidate the function of these receptors.

### Significance of α7-nAChRs in Retinal Bipolar Cells

Located in the midretina, bipolar cells relay visual signals from photoreceptors to ganglion cells, the input and output neurons of the retina, respectively. Bipolar cell types are identified by their axon terminal ramification patterns in the inner plexiform layer (IPL), where synaptic interactions among bipolar, amacrine, and ganglion cells occur. Each type of bipolar cell is thought to extract different components of visual signals from photoreceptors, such as color and motion, and pass these components to discrete retinal circuits, forming parallel processing pathways.^62^–^64^ Signal transmission from bipolar cells to retinal ganglion cells is then modulated either by feedback or feedforward effects of amacrine cells.

In addition to unique axonal ramification patterns, bipolar cell types exhibit molecular and physiological differences. Wässle et al.^2^ established that each type of bipolar cell can be recognized by unique markers, such as antibodies or transgenic mouse lines. Furthermore, Shekhar et al.^4^ used single-cell transcriptomics to show that each type of bipolar cell expresses different sets of genes. They further found that various kinds of voltage-gated channels, receptors, transcription factors, and adhesion molecules are uniquely expressed by each type of bipolar cell. Furthermore, we found that each type of cone bipolar cell exhibits unique temporal properties in response to sinusoidal light stimuli.^14^,^15^ Similarly, Baden et al.^12^ and Borghuis et al.^65^ used fluorescence imaging tools to show that temporal responses and spike generation differ among bipolar cells ramifying in different layers of the IPL. These reports suggest that each bipolar cell responds differently to certain types of images, and thus, transfers diverse kinds of visual signals to different neural networks in the IPL.

Our observation that a subset of retinal bipolar cells express α7-nAChRs further adds to the diversity of factors modulating bipolar cell output. α7-nAChRs exhibit high calcium permeability, which excites cells and could regulate neurotransmitter release from axon terminals.^66^ It has been demonstrated that glutamatergic neurons throughout the central nervous system express α7-nAChRs, which facilitate glutamate release from presynaptic axon terminals.^34^–^36^,^38^,^67^ Furthermore, presynaptic α7-nAChRs facilitate other types of neurotransmitter release^37^,^68^,^69^ through direct passage of calcium into the presynaptic terminal.^70^,^71^ It may also be possible that these channels activate nearby voltage-gated calcium channels.^68^ The expression of α7-nAChRs in glutamatergic bipolar cells therefore suggests a similar role in facilitating glutamate release to postsynaptic neurons.

### Implications for the Role of α7-nAChR-Expressing Bipolar Cells in the Retinal Neural Network

GABAergic and glycinergic amacrine cells have been shown to modulate bipolar cell outputs via presynaptic inhibition, thereby increasing bipolar cell functional diversity.^17^ Conversely, presynaptic bipolar cell expression of α7-nAChRs suggests another possible mechanism for directly altering bipolar cell outputs via feedback amacrine cell excitation. SACs, which release acetylcholine in response to preferred directional stimulation, are the only cholinergic neurons in the retina.^72^ Thus, neurons that receive acetylcholine inputs, such as α7-nAChR-containing bipolar cells, may be important for retinal motion detection, especially given previous reports demonstrating that SACs and DSGCs receive synaptic inputs from particular types of bipolar cells, including type 5 bipolar cells.^3^,^73^ Previous reports, however, demonstrate that bipolar cells themselves do not exhibit direction selectivity.^73^–^76^ These results have led to a consensus that bipolar cells do not play a major role in retinal direction selectivity.

Nevertheless, this assumption was recently challenged via connectomic data that revealed how varying bipolar cell synaptic partners, as well as the arrangement of bipolar cell types within motion circuits, may contribute to retinal motion detection. These studies revealed that different types of bipolar cells not only carry visual signals to different IPL layers, but also make divergent synaptic connections to postsynaptic neurons. For example, multiple sets of type 5 bipolar cells ramify near the ON-ChAT band within sublamina 3^13^; however, only one subset of type 5 provides synaptic inputs to DSGCs. Conversely, XBC-type 5 cells do not provide synaptic inputs to DSGCs.^3^ In the case of SACs, different types of bipolar cells provide synaptic inputs to various locations along their dendrites.^51^,^77^,^78^ Our results indicate that α7-nAChRs are expressed by type 1, 2, 4, and 7 bipolar cells. Interestingly, type 4 bipolar cells are the main “OFF” inputs to ON/OFF DSGCs.^3^ Furthermore, the other three types of α7-nAChR-expressing bipolar cells provide synaptic inputs to SACs at proximal dendrites.^51^,^77^,^78^ In conjunction with connectomic studies, our results imply that particular types of bipolar cells play a pivotal role in motion detection.

SACs release both GABA and acetylcholine in response to a moving object. The contribution of acetylcholine to retinal motion detection is highly debated, with some groups suggesting acetylcholine contributes almost half of DSGC excitation while other groups find that glutamate instead predominantly provides DSGC excitation.^28^–^30^,^75^,^79^–^81^ Although the functional outcome of α7-nAChRs in bipolar cells to direction selectivity in the third-order neurons will require future investigation, our new findings may contribute to the elucidation of acetylcholine involvement in motion detection circuit.
